# Expression of leptin and its long form receptor at the porcine maternal-fetal interface: contrasting healthy and arresting conceptus attachment sites during early and mid-pregnancy

**DOI:** 10.1186/1477-7827-12-91

**Published:** 2014-09-23

**Authors:** Ashley Kerr, Rami T Kridli, Kasra Khalaj, Jocelyn M Wessels, Ann Hahnel, Chandrakant Tayade

**Affiliations:** Department of Biomedical Sciences, University of Guelph, Guelph, Ontario Canada; Department of Animal Production, Jordan University of Science and Technology, Irbid, Jordan; Department of Biomedical and Molecular Sciences, Queen’s University, Kingston, Ontario Canada; Department of Obstetrics and Gynecology, McMaster University, Hamilton, Ontario Canada

**Keywords:** Pigs, Gestation, Endometrium, Chorioallantoic membrane, PCR, Western blotting, Immunohistochemistry

## Abstract

**Background:**

It is well established that spontaneous conceptus loss in swine is associated with an imbalance of both angiogenic and immunological factors. Leptin (LEP), a metabolic hormone, has also been implicated in the promotion of angiogenesis. In this study, LEP and its long form receptor (OB-Rb) were evaluated during porcine pregnancy to assess their basal level of expression and their potential role in conceptus development.

**Methods:**

Expression and secretion of LEP and OB-Rb were quantified in the endometrium of non-pregnant (n = 5), and in the endometrium and chorioallantoic membrane (CAM) of pregnant sows (parity 2 to 5) at gestational days (gd) 20 (n = 8) and 50 (n = 8). Data were analyzed by a 3-way ANOVA testing the effects of conceptus health, tissue type and gestational day.

**Results:**

Leptin and *OB-Rb* transcripts were significantly higher (P < 0.05) in pregnant than in non-pregnant sows. Significantly greater *LEP* (P < 0.001) was detected in the endometrial tissue at gd20 compared with gd50. At the protein level, the lowest LEP expression (P < 0.01) was detected in the CAM at gd50, while OB-Rb protein was significantly lower (P < 0.01) at gd50 in the CAM than in the endometrium collected from gd20 and gd50 conceptus attachment sites. Immunofluorescence staining confirmed the expression of these proteins at both gestational days and in both tissue types.

**Conclusions:**

Changes in the expression patterns of LEP and OB-Rb between gd20 and gd50 suggest a role for the LEP/OB-R complex at the early stages of porcine pregnancy, possibly affecting the attachment process. Further mechanistic studies are warranted to understand the specific role of leptin in porcine pregnancy.

## Background

Prenatal embryonic/fetal loss is a major concern for North American pork producers with approximately 30 to 45% of the conceptuses lost during gestation (gestational length is 114 days) [1]. Two waves of spontaneous conceptus loss are documented: a primary loss of approximately 20-30% during the time of peri-attachment (around gestational day (gd) 15–20) and a second loss of 10-15% during mid-gestation (around gd50) [1–8]. Understanding the physiological and molecular mechanisms behind these losses may help increase litter size, thus positively impacting the economic production of commercial swine.

Numerous factors contribute to early conceptus losses [4, 9]. Previous studies from our laboratory have demonstrated that conceptus losses are associated with an imbalance of mRNA transcripts for multiple angiogenic and inflammatory cytokines [10–12] as well as factors that can regulate mRNA stability such as the tristetraprolin family members (Khalaj et al., unpublished data). Arresting conceptuses are recognized by reduced size, weight, and vasculature at the sites of attachment, when compared to their healthy, more robust littermates [10]. The reduction in vascularization has been shown to be associated with a decreased production of vascular endothelial growth factor (VEGF) transcripts [6, 10]. These findings suggest a correlation between abnormal angiogenesis at the maternal-fetal interface and fetal loss.

An angiogenesis-related protein of interest that has been linked to reproductive function and embryonic development is leptin (LEP) [13]. Leptin, a 16 kDa, non-glycosylated polypeptide, is not only thought to have a local autocrine and immunomodulatory role in immune function and angiogenesis, but is also involved in energy metabolism, satiety, and reproduction [14, 15]. The biological actions of LEP depend greatly on the presence of LEP receptors (OB-R) for which there are six identified isoforms: OB-Ra, OB-Rb, OB-Rc, OB-Rd, OB-Re, and OB-Rf [16]. The long form of the LEP receptor (OB-Rb, contains tyrosine residues) is of particular interest due to its role in mediating LEP signaling through the cell membrane [16]. The short isoforms are primarily involved in leptin transport across the blood–brain barrier [16]

As previously mentioned, appropriate blood supply to the developing conceptus is essential for a successful pregnancy. The binding of LEP to OB-Rb within endothelial cells has been linked to the activation of the JAK/STAT3 molecular pathway which promotes endothelial tube formations and corneal neovascularization *in vitro* [13, 17, 18] and has also been associated with the up-regulation of VEGF transcription [19]. These particular findings suggest that the role of LEP in the activation of JAK/STAT3 pathway may enhance embryonic attachment and angiogenesis at the maternal-fetal interface.

Different patterns of uterine *LEP* and *OB-Rb* transcription have been reported during porcine pregnancy. Endometrial expression of *LEP* at gd30-32 is significantly increased in comparison with gd14-16 [20]. The same laboratory reported no differences in *OB-Rb* expression between these stages of pregnancy [21]. Besides the uterus, both *LEP* and *OB-Rb* have been detected at a higher level in the ovary during early pregnancy compared with cycling sows pointing to a role during gestation [22]. In addition, *OB-Rb* transcripts have been noted to be differentially expressed in the implantation and the inter-implantation sites of pregnant mice, suggesting that the LEP/OB-R complex may be a regulator in the implantation process [23]. Leptin and OB-Rb gene and protein expression at the maternal-fetal interface in swine has yet to be examined in healthy and arresting attachment sites during the two waves of spontaneous conceptus losses. Therefore, the aim of this study was to determine if LEP and OB-Rb are expressed during early (gd20) and mid-gestation (gd50) of porcine pregnancy and whether their expression is influenced by conceptus health.

## Methods

### Porcine tissue sample collection

Pathogen-free Yorkshire sows (n = 21) from Arkell Swine Research Station (University of Guelph, Guelph, ON, Canada) were used for this study. Sows (parity 2 to5) were artificially bred through means of a sponge-tipped catheter using pooled boar semen. Tissue samples were collected from non-pregnant sows in the diestrus stage of the estrous cycle (n = 5), and pregnant animals at gd20 (n = 8, peri-attachment) and gd50 (n = 8, mid-gestation). Animal handling and euthanasia protocols were approved by the University of Guelph Animal Care Committee (Animal utilization protocol #10R061). Immediately after slaughter, the reproductive tracts were collected at the University of Guelph abattoir and transported to the laboratory on ice. Uteri were opened longitudinally along the anti-mesometrial side to remove embryos from the attachment sites. The embryos of each sow were individually categorized as healthy or arresting based on visual assessment of vascularization and color of the membrane attachment areas, as well as fetal length and weight as previously described [6, 10]. Any conceptuses that had extreme resorption or a debatable health status were removed from the study. At each attachment site, endometrial tissue on the maternal side was collected separately from the corresponding chorioallantoic membrane (CAM) from the fetal side. Within a litter, each collected tissue was treated as an individual attachment site and not pooled. For non-pregnant samples, endometrial tissue was removed from the mesometrial side at random. All samples were rinsed with PBS prior to storage. Each tissue was divided into multiple samples: samples for RNA isolation or protein extraction were frozen immediately and stored at −80°C, while samples for immunoflouresence were fixed in paraformaldehyde and embedded in a paraffin mold.

### RNA isolation from endometrial and CAM samples

Total RNA extraction on all collected tissues was performed using RNeasy mini kits (Qiagen, Mississauga, ON, Canada) as per the manufacturer’s instructions. RNA concentration was determined using the Gene Quant pro RNA/DNA calculator (Biochrom Ltd, Cambridge, UK). RNA quality was assessed by evaluating the A260/A280. All samples were within purity range (2.0 +/− 0.25). RNA was immediately frozen at −80°C for future use.

### Reverse transcription

cDNA synthesis was performed using First-Strand cDNA Synthesis Kit as per manufacturer’s instructions (GE Healthcare Bio-Science Inc. Baie d’Urfe, QC, Canada). Briefly, 20 μL of diluted RNA (concentrations ranged from 224 to 890 ng/μL) was heated at 65°C for 10 minutes in the GeneAMP polymerase chain reaction (PCR) System 2700 (Applied Biosystems, Foster, CA, USA). Once complete, 11 μL of the bulk first strand cDNA reaction mix, 1 μL of poly (dT) primer, and 1 μL of DTT solution was added. The reaction was incubated at 37°C for 60 minutes. The cDNA concentration was measured using a Gene Quant Pro RNA/DNA calculator (Biochrom Ltd, Cambridge, UK). All cDNA products were stored in −20°C for subsequent real time PCR use.

### Quantitative real-time PCR

Primers targeting the genes of interest (LEP and OB-Rb) were designed from the electronic nucleotide database, GenBank, using Primer 3 software (http://biotools.umassmed.edu/bioapps/primer3_www.cgi). To test the primers and to optimize their efficiency, aliquots of cDNA from all tissues were pooled and used as a template. Information on the primers for each gene of interest is provided in Table 1. Quantitect SYBR Green I PCR mix kit was used to optimize primer efficiency (Qiagen, Mississauga, ON, Canada) in a capillary-based real time PCR system (LightCycler, Roche Diagnostics, Laval, QC, Canada). Genes quantified in all experimental samples were run in duplicate using the 384 PCR plate of the LightCycler 480 (Roche Diagnostics, Laval, QC, Canada) as described by Wessels et al. [24]. Data was expressed as a ratio of *LEP* or *OB-Rb* mRNA relative to *β-actin* (*ACTB)* mRNA. Sample numbers used in the various analyses are shown in Table 2. The difference in sample size is primarily due to a lower number of arresting conceptuses available during gestation.

**Table 1:** **List of primers used for β-actin (ACTB), leptin (LEP) and the long form leptin receptor (OB-Rb)**

Gene	Primers^1^(5′to3′)	Product size (bp)	Designed from GenBank accession no.
*LEP*	F: TTCTCTCTCGCTCCGCTAAG	156	AF026976.2
	R: GAAGGAAGACGTTGGTGGAA		
*OB-Rb*	F: TTAGTGACCAACGCAGCAGT	243	AF092422
	R: AGGCCTGGGTTTCTATCTCC		
*ACTB*	F: ACGTGGACATCAGGAAGGAC	210	DQ452569.1
	R: ACATCTGCTGGAAGGTGGAC		

^1^F = forward and R = Reverse.

**Table 2:** **The number of samples of each conceptus type used in the polymerase chain reaction (PCR), Western Blotting (WB) and immunofluorescence (IF) analyses** ^**1**^

	NP	Gestational day 20				Gestational day 50			
		Endometrium		Chorioallantoic membrane		Endometrium		Chorioallantoic membrane	
		H	A	H	A	H	A	H	A
*PCR samples (No.)*	5	30	19	26	20	29	6	27	4
*WB samples (No.)*	-	6	6	6	6	6	6	6	6
*IF samples (No. of sections from different animals)*	-	3	3	3	3	3	3	3	3

^1^NP = non-pregnant; H = healthy conceptus attachment site; A = arresting conceptus attachment site.

### Western immunoblotting for LEP and OB-Rb

Tissue samples were removed from the −80°C freezer, thawed on ice and weighed (30 mg) into Eppendorf tubes. For each sample, protease inhibitor (2 μL Aprotonin, Sigma Aldrich Co., St Louis, MO) and 200 μL of phosphate buffered saline (PBS) were added before homogenization on ice for 1 minute. Samples were then centrifuged (6000 × g) at 4°C for 15 minutes and supernatants were harvested. Protein concentration was quantified using the Bradford method. Aliquots of the protein samples were diluted to a final concentration of 2 μg/μL and stored at −80°C until analyzed for specific proteins. Six samples from each of the tissues were used for protein extraction and Western Blotting.

Leptin and OB-Rb proteins were quantified by Western Blot. In brief, 10 μg protein samples were denatured at 100°C for 5 minutes. Samples were then loaded into appropriate wells of 4-20% pre-cast mini-PROTEAN TGX gels (12 wells/20uL) (Bio-Rad Laboratories, Mississauga, ON) and separated by electrophoresis at140 V for 1 hour. Proteins were transferred onto nitrocellulose membrane using 100 V for 2 hours. The membrane was blocked with 5% skimmed milk (reconstituted in tris-buffered saline containing Tween 20 (TBS-T)) for 1 hour at room temperature. The membrane was then incubated in primary antibody [Pierce Leptin polyclonal rabbit (PA1-052, Thermo Fisher Scientific, Waltham, MA), or OB-R (H-300) rabbit polyclonal IgG (sc-8325, Santa Cruz Biotechnologies, Inc., Dallas, TX)] diluted in skimmed milk (1 μg/mL for LEP and 2.5 μg/mL for OB-Rb) at 4°C overnight. After washing with TBS-T, the membrane was incubated in the secondary antibody diluted in skimmed milk [0.01 μg/mL for LEP and 0.16 μg/mL for OB-Rb; Anti-rabbit IgG (whole molecule)-peroxidase antibody produced in goat (Sigma Aldrich Co., St Louis, MO)] for 1 hour at room temperature. The membrane was then washed and imaged using an electrochemiluminescence kit (Bio-Rad Laboratories, Mississauga, ON) and a Konica Minolta SRX-101A X-ray film developer (Konica Minolta Ltd., Mississauga, ON). Leptin and OB-Rb were run on separate membranes. After imaging, all membranes were stripped using Restore Western Blot Stripping Buffer (Thermo Scientific, Rockford, IL) and re-probed for ACTB, a loading control for both proteins. The same protocol was followed for ACTB except that a primary antibody pre-conjugated with horseradish perioxidase [HRP; anti-beta Actin antibody (HRP; mAbcam 8226, Abcam PLC, Cambridge, England)] was used (0.2 μg/mL). The X-ray images were scanned at 600 dpi (greyscale) and analyzed using ImageJ software (NIH, Bethesda, MD) to obtain densitometry values as a ratio to ACTB from each corresponding blot.

### Staining: immunofluorescence (IF) and hematoxylin and eosin (H&E)

Formalin-fixed, paraffin-embedded endometrial and CAM tissues were sectioned at 7 μm thickness and mounted on Superfrost Plus microscope slides (Fisher Scientific, Ottawa, ON, Canada). Tissue sections were then deparaffinized with xylene and rehydrated with ethanol. A 0.2% hydrogen peroxide solution (diluted in methanol) was applied to sections for 20 minutes to block endogenous peroxidase activity. Slides were then rinsed with PBS, boiled in a sodium citrate buffer (pH 6.0) and allowed to cool for 45 minutes at room temperature. Sections were rinsed with PBS then blocked with 1.0% bovine serum albumin (BSA) in PBS for 1 hour at room temperature. Primary antibody for LEP and OB-Rb [0.5 μg per section in 0.1% BSA in PBS (10 ng/μL); the same primary antibodies used in Western Blotting were used in IF] were applied to sections and incubated overnight at 4°C. Serial antibody dilutions were tested before optimization. Control sections were incubated with rabbit immunoglobulin [ab37415, Abcam PLC, Cambridge, England; 0.5 μg per section in 0.1% BSA in PBS (10 ng/μL)] as a negative control. Following incubation, sections were rinsed in PBS before the secondary antibody [Alexa Fluor 594 donkey anti rabbit IgG (A21207, Invitrogen, Carlsbad, CA); 0.15 μg per section in PBS (3 ng/μL)] was placed on each tissue section, including the negative controls, and allowed to incubate for 1 hour at room temperature. Slides were protected from light from this point onward. Sections were then rinsed with PBS, mounted using Aqua Poly/Mount mounting media (Polysciences, Inc., Warrington, PA, USA) and imaged (200x) using xenon light microscope equipped with a FITC filter (Leica Microsystems AG, Wetzlar, Germany) at a 2.5 second exposure time. The same sections above were subsequently stained with H&E. As often as possible, the same area captured following the IF staining was imaged after H&E staining in order to allow for a direct comparison.

### Statistical analysis

For real-time PCR data, a Grubbs test was performed on each experimental group to remove any outliers (http://graphpad.com/quickcalcs/Grubbs1.cfm). Statistical analyses of quantitative real-time PCR and Western Blot for both LEP and OB-Rb were conducted using Sigma Stat 3.5 software (Systat Software Inc., Chicago, IL, USA). All data were logarithmically transformed to attain normality before statistical analyses. Proper tests were set within the software to ensure that the assumptions of ANOVA are met. With respect to real-time PCR data, NP data were compared to data from gd20 endometrial tissue samples only, while all CAM and endometrium samples across both gestational days were compared with each other. The effect of conceptus health, tissue type and gestational day on transcript and protein expression was tested using a three-way analysis of variance for repeated measures. When a significant interaction was detected, only the interaction was presented while ignoring the main effect means. For the three-way ANOVA, a Holm-Sidak post hoc test was used. A p-value < 0.05 was considered significant.

## Results

### *LEP*expression

**Figure 1:**
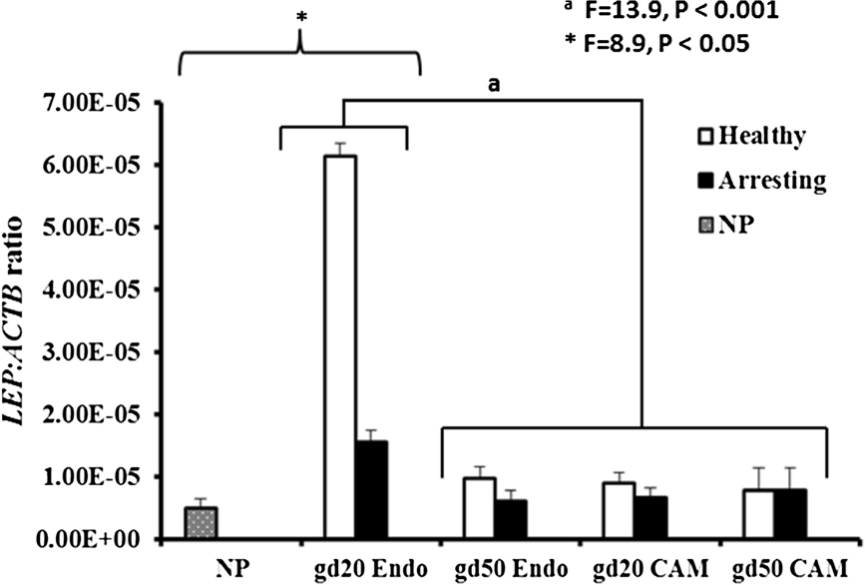
**Leptin (** ***LEP*** **) mRNA expression level comparison between gestational days (gd) 20 and 50 healthy and arresting conceptus attachment sites.** Relative mRNA expression levels of *LEP* in the endometrium of non-pregnant (NP; grey filled bar), in comparison with endometrial (Endo) and chorioallantoic membrane (CAM) tissues of healthy (white-filled bars) and arresting (black-filled bars) attachment sites at gestational day (gd) 20 and gd50. ^*^ *LEP* expression in NP samples was compared with gd20 endometrial tissues only (P < 0.05). Conceptus health did not affect *LEP* expression. ^a^Significant tissue type by gd interaction existed (P < 0.001). The greatest *LEP* expression was detected in endometrial samples collected from conceptus attachment sites at gd20 when compared with the remaining tissues on both gestational days. Transcript abundance was quantified by real-time PCR and normalized as a ratio to β-actin (ACTB). Histogram bars represent group means plus standard error.

Leptin transcripts were found to significantly increase (P = 0.015) in early pregnancy (gd20) endometrium compared to the non-pregnant tissues. Overall, *LEP* expression across samples did not differ between healthy and arresting conceptus attachment sites (Figure 1). There was a significant tissue type by gestational day interaction (P < 0.001) in *LEP* expression. The greatest *LEP* expression was detected in endometrial samples collected from conceptus attachment sites at gd20 when compared with endometrial samples at gd50 as well as CAM samples collected at gd20 and gd50 (Figure 1), regardless of conceptus health. As pregnancy progressed into mid-gestation (gd50), there was a significant decrease in *LEP* expression in the endometrium with no change in *LEP* transcripts in the CAM for both healthy and arresting conceptus attachment sites (Figure 1).

### *OB-Rb*expression

**Figure 2:**
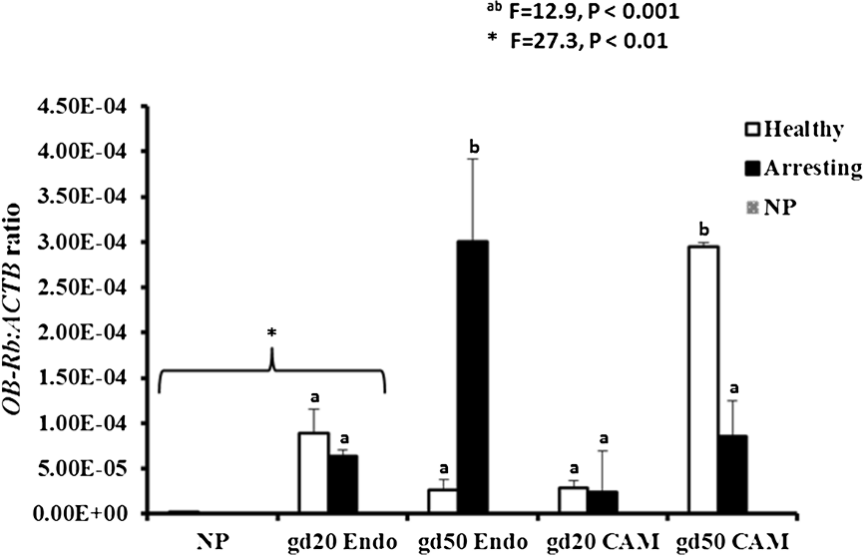
**Leptin receptor (** ***OB-Rb*** **) mRNA expression level comparison between gestational days (gd) 20 and 50 healthy and arresting conceptus attachment sites.** Relative mRNA expression levels of leptin receptor (*OB-Rb*) in the endometrial tissue of non-pregnant (NP; grey filled bar), in comparison with endometrial (Endo) and chorioallantoic membrane (CAM) tissues of healthy (white-filled bars) and arresting (black-filled bars) attachment sites at gestational day (gd) 20 and gd50. *OB-Rb expression in NP samples was compared with gd20 endometrial tissues only (P < 0.05). A three-way interaction existed between conceptus health, tissue type and gd (P < 0.001). The expression of *OB-Rb* was greater in gd50 CAM from healthy and endometrium form arrested attachment sites than all other tissues at both gestational days. ^ab^Bars with different superscripts are significantly different (P < 0.001). Transcript abundance was quantified by real-time PCR and normalized as a ratio to β-actin (ACTB). Histogram bars represent group means plus standard error.

The *OB-Rb* transcripts were found in the non-pregnant and pregnant endometrium at gd20 and gd50, as well as the CAM at gd20 and gd50. At day 20 of pregnancy, *OB-Rb* expression levels in the endometrium were significantly elevated (P < 0.01) in healthy and arresting conceptus attachment sites in comparison to the non-pregnant samples. There was a significant (P < 0.001) three-way interaction (conceptus health by tissue type by gestational day) with respect to *OB-Rb* expression (Figure 2). The expression of *OB-Rb* was greater in gd50 CAM from healthy as well as in endometrium from arresting attachment sites than all other tissues. The lowest *OB-Rb* expression was observed in the endometrium samples from healthy attachment sites at gd50 and the CAM from healthy and arresting attachment sites at gd20.

### LEP and OB-Rb protein quantification

**Figure 3:**
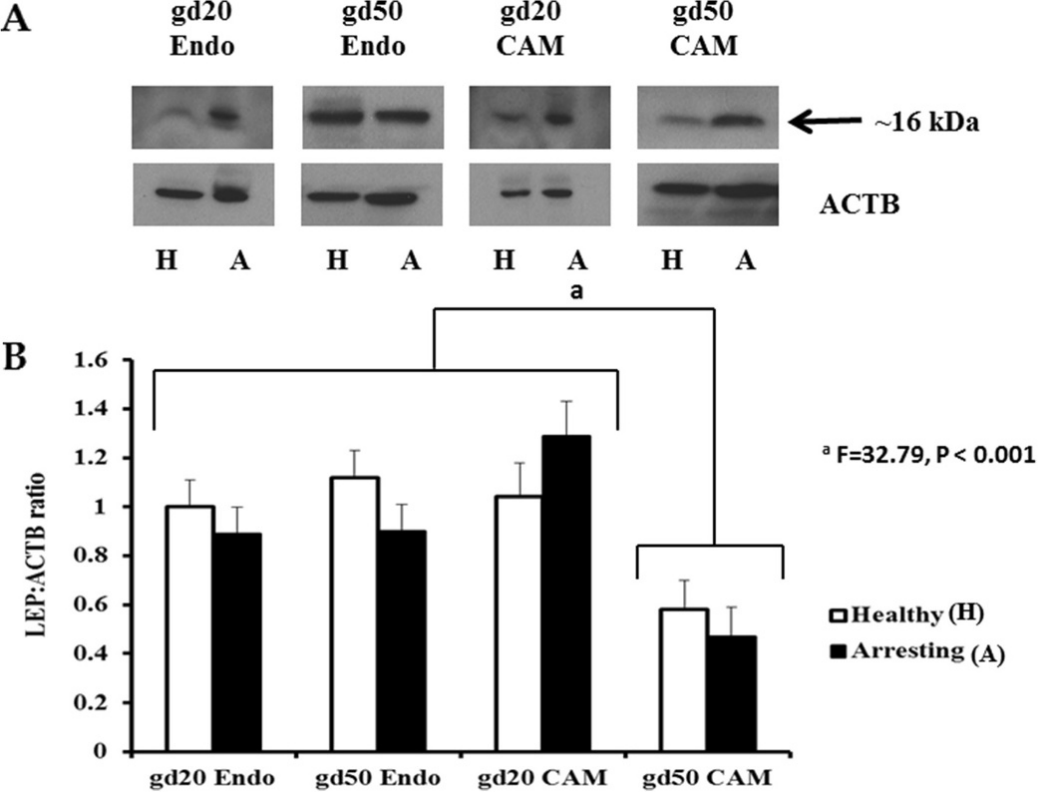
**Leptin (LEP) protein expression at gestational days (gd) 20 and 50 healthy and arresting conceptus attachment sites. A**: Western Blotting images of LEP protein expression levels in the endometrial (Endo) and chorioallantoic membrane (CAM) tissues of healthy and arresting attachment sites at gestational day (gd) 20 and gd50. The band in the 16 kDa range was quantified relative to the expression of β-actin (ACTB). **B**: Histogram represents densitometry values of relative LEP protein expression levels in the Endo and CAM tissues of healthy (white-filled bars) and arresting (black-filled bars) attachment sites at gd20 and gd50. Statistical comparisons were made based on the gd by tissue type interaction. The letter “a” denotes significantly lower (P < 0.001) LEP production in gd50 CAM samples than the remaining tissues at both gestational days. Histogram bars represent group means plus standard error.

**Figure 4:**
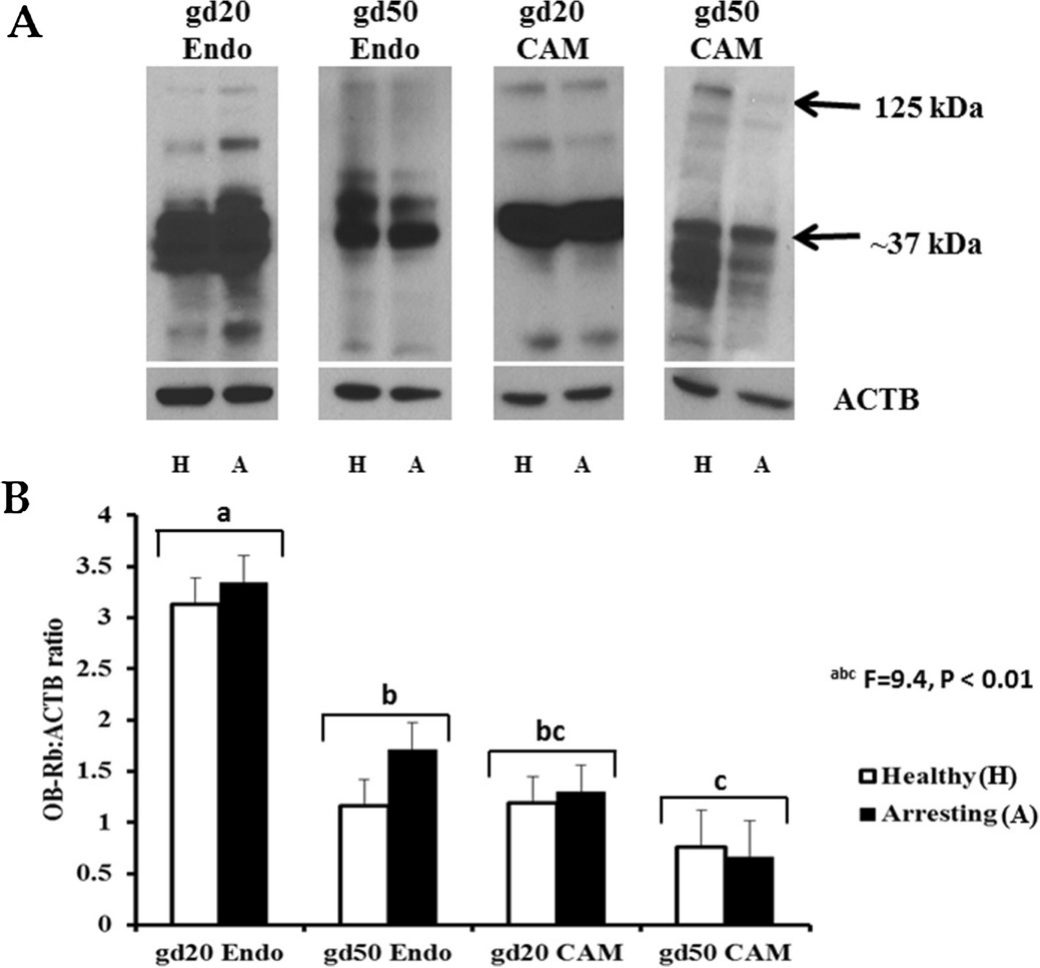
**Leptin receptor (OB-Rb) protein expression at gestational days (gd) 20 and 50 healthy and arresting conceptus attachment sites. A**: Western Blotting images of relative OB-Rb protein expression levels in the endometrial (Endo) and chorioallantoic membrane (CAM) tissues of healthy and arresting attachment sites at gestational day (gd) 20 and gd50. Only the top band (~125 kDa range) was quantified relative to the expression of β-actin (ACTB). **B**: Histogram represents densitometry values of relative OB-Rb protein expression levels in the Endo and CAM tissues of healthy (white-filled bars) and arresting (black-filled bars) attachment sites at gd20 and gd50. Statistical comparisons were made based on the gd by tissue type interaction. ^abc^Bars (pulled together from healthy and arresting attachment sites) with different superscripts are significantly different (P < 0.01). The OB-Rb protein production was more pronounced in the endometrium than the CAM, and again more at gd20 than gd50. The lowest OB-Rb production was observed in the gd50 CAM samples compared with the remaining tissues. Histogram bars represent group means plus standard error.

Western blotting showed that LEP was produced at both the maternal and fetal sides on both gestational days 20 and 50 (Figure 3A and 3B). No differences in LEP expression were observed between healthy and arresting conceptus attachment sites on either gestational day. The production of LEP was similar in the CAM and endometrial tissue samples, however being significantly influenced by gestational day (P < 0.01) and the tissue type by gestational day interaction (P < 0.001). The LEP protein was more predominant at gd20 but its production was affected by tissue type. CAM from gd50 samples had the least amount (P < 0.001) of LEP protein when compared with endometrial samples at gd50 as well as CAM and endometrial samples collected at gd20 (Figure 3B) regardless of conceptus health.Leptin receptor quantification revealed multiple bands representing the different isoforms of the receptor (Figure 4A). As we are interested in the long form receptor, we quantified the largest band that was obtained (approximately 125 kDa). However, the band with the greatest signal intensity was detected around the 37 kDa range (Figure 4A). Similar to LEP, no differences in the OB-Rb production were noted between healthy and arresting conceptus attachment sites on either gestational day (Figure 4B). However, in the case of OB-Rb, production of the protein significantly differed between tissue types (P < 0.001), gestational day (P < 0.001) and their interaction (P < 0.01). The OB-Rb protein was produced at a higher level in the endometrium than the CAM, and at gd20 compared with gd50.Taking the interaction into consideration, the greatest amount of OB-Rb protein was produced in the endometrium collected from conceptus attachment sites at gd20, and the lowest was produced in CAM collected from conceptus attachment sites at gd50 with the remaining samples having intermediate OB-Rb protein production.

### Localization of LEP and OB-Rb at the fetal and maternal sides

**Figure 5:**
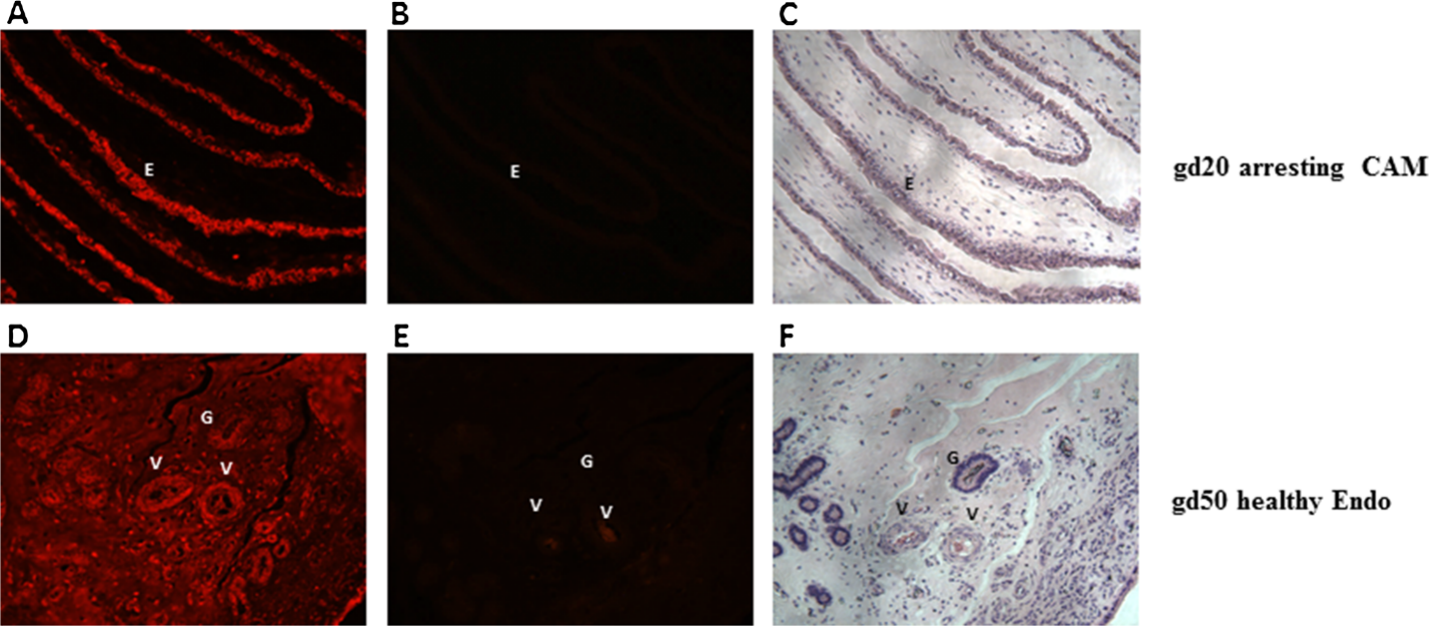
**Localization of the leptin (LEP) protein at gestational days (gd) 20 and 50 in the chorioallantoic membrane (CAM) and endometrium (Endo) of the porcine maternal-fetal interface.** Representative immunofluorescence images of LEP localization in the endometrium and CAM. No differences were observed among tissues regardless of conceptus health status and gestational day. Images **A**, **B** and **C** show an experimental sample (with glandular staining), a negative control (rabbit immunoglobulin), and a hematoxylin stain of an endometrial tissue section, respectively. Images **D**, **E** and **F** show an experimental sample (CAM staining), a negative control (rabbit immunoglobulin), and the hematoxylin stain of a CAM tissue section, respectively. “G” indicates gland, “V” indicates blood vessel and “E” indicates epithelium. Magnified at 200x.

**Figure 6:**
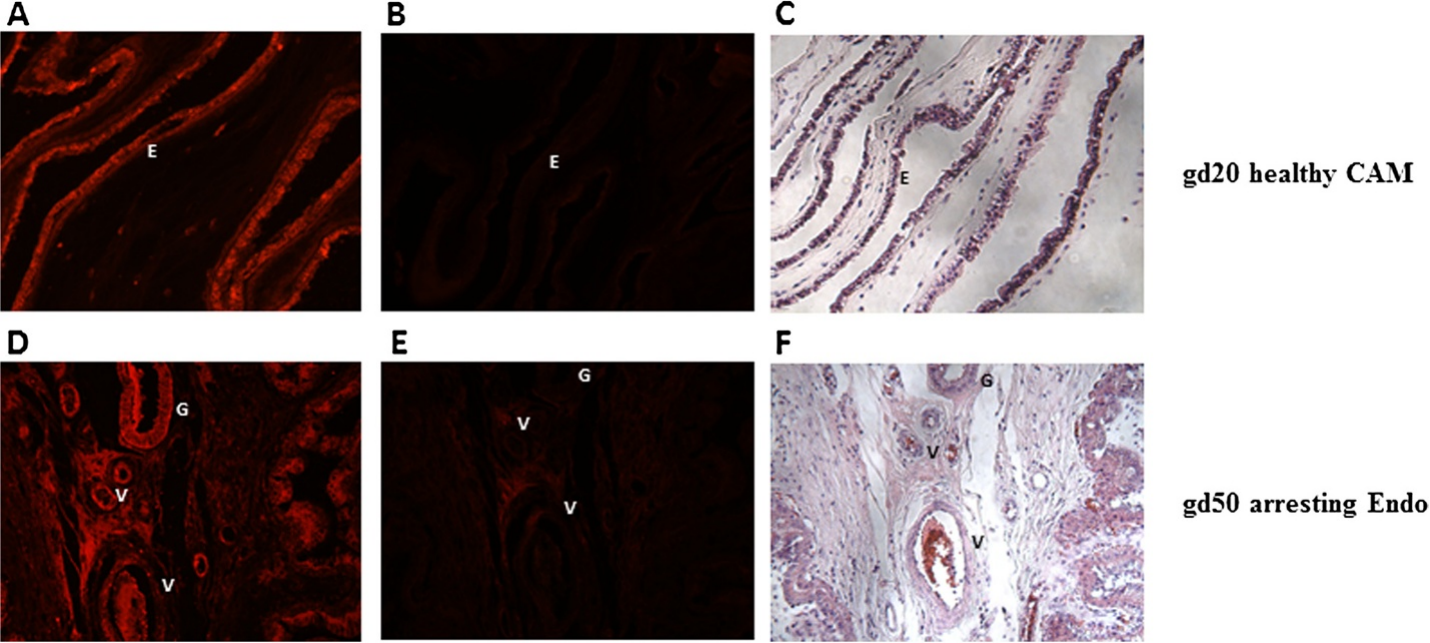
**Localization of the leptin receptor (OB-Rb) protein at gestational days (gd) 20 and 50 in the chorioallantoic membrane (CAM) and endometrium (Endo) of the porcine maternal-fetal interface.** Representative immunofluorescence images of OB-Rb localization in the endometrium and CAM. No differences were observed among tissues regardless of conceptus health status and gestational day. Images **A**, **B** and **C** show an experimental sample (with glandular staining), a negative control (rabbit immunoglobulin), and a hematoxylin stain of an endometrial tissue section, respectively. Images **D**, **E** and **F** show an experimental sample (CAM staining), a negative control (rabbit immunoglobulin), and the hematoxylin stain of a CAM tissue section, respectively. “G” indicates gland, “V” indicates blood vessel and “E” indicates epithelium. Magnified at 200x.

Figures 5 and 6 show representative sections of immunofluorescence staining for LEP and OB-Rb, respectively, in endometrium and CAM. Leptin and OB-Rb proteins were highly expressed on both gestational days in both maternal and fetal compartments. Intense stromal as well as glandular and endothelial LEP staining was observed in the endometrial sections and in the fetal CAM. Similarly, in the case of OB-Rb, staining was observed in the endometrial glands, stroma and blood vessels and in CAM from the fetal side. Visually, we did not observe any noticeable difference in the production patterns for either protein regardless of conceptus health and gestational day. The positive staining described was compared to the control sections presented in Figures 5 and 6.

## Discussion

The overall results of the current study show that LEP and OB-Rb transcripts and proteins are expressed and produced in the conceptus attachment sites while being influenced by the type of tissue as well as the two periods of spontaneous conceptus loss (gd20 and 50). The fact that *LEP* and *OB-Rb* mRNA transcripts are greater in the pregnant than the non-pregnant endometrium points to a role during early porcine pregnancy. Previous studies have indicated that LEP plays a role in fetal growth and development towards the end of gestation, in addition to helping the fetus transition from intrauterine to extrauterine life [25]. Yet, the exact role that LEP has during early pregnancy is still unclear.

Leptin and OB-Rb mRNA and protein have been shown to be expressed and produced in the placenta of a wide range of species including humans, pigs, baboons, sheep, mice and rats [25]. Additionally, increased LEP production was reported in the anterior pituitary of early pregnant sows (gd 14–16) over mid pregnancy (gd 30–32) or non-pregnant cycling sows [26]. The *LEP* mRNA expression in the current study was influenced by the tissue type by gestational day interaction, while the *OB-Rb* expression was affected by conceptus health by tissue type by gestational day interaction. The detected interactions indicate that the expression patterns of both *LEP* and *OB-Rb* change based on tissue and with the advancement of gestation. Increased *LEP* was observed in the endometrial tissue of conceptus attachment sites at gd20 compared with the remaining tissues and more so in healthy than in arresting conceptus attachment sites. Gu et al. [27] reported greater *LEP* expression during the early gestation of Meishan than Yorkshire gilts. The authors linked this increased *LEP* expression with the higher prolificacy of Meishan pigs. At gd50, *OB-Rb* was greater in the endometrium of arresting and CAM of healthy conceptus attachment sites than the remaining tissues. Increased *OB-Rb* in the porcine CAM has been reported as pregnancy progressed [21]. An increase in OB-Rb and OB-Rb signaling over pregnancy may be important for pregnancy maintenance [28]. This suggests that a reduced amount of LEP lowers receptor signaling in the endometrium, creating a uterine environment that may be unable to maintain healthy developing conceptuses. The current results also support this hypothesis, as *LEP* transcripts, although not significant, tend to be expressed at lower levels in the endometrium of arresting sites compared to healthy. Similarly, Simon et al. [29] reported lower LEP levels in arresting human blastocysts compared to those which were healthy, further implicating LEP/OB-Rb in embryonic loss. A reduction or withdrawal of LEP during the pregnancy can result in embryonic loss [30]. As such, areas within the uterus with a reduction in *LEP* gene expression seem to lead to an environment where fetal demise can ultimately occur.

Both LEP and OB-Rb proteins were quantified by Western Blotting and localized through staining. The glandular and stromal localization of LEP and OB-Rb proteins was similar to what was previously reported [20, 21]. Although similar protein production patterns were observed (Figures 5 and 6), our findings were the first to show that these proteins are produced in healthy and arresting attachment sites at both gd20 and gd50.

The primary antibody used to quantify leptin receptors in this study targets both the short and long forms. Unlike other studies that detected a band for the long form (OB-Rb) in the 170 kDa range [26], the largest OB-Rb bands we detected fell in the 125 kDa range. In the current study, LEP and OB-Rb proteins did not vary between healthy and arresting attachment sites. Both proteins were affected by the interaction between tissue type and gestational day. The lowest level of LEP protein was found in the CAM at gd50, while OB-Rb was lowest at gd50 in the CAM as compared to the gd20 and 50 endometrium; both irrespective of conceptus health. Thus, because LEP and OB-Rb are lower at the fetal side (CAM) at gd50, the LEP/OB-R complex could potentially be critical for survival and, possibly, attachment of the early porcine embryo. The variation in OB-Rb gene expression and protein production across tissues and gestational days is most likely related to changes in their sensitivity to LEP. A similar finding was reported by Smolinska et al. [21] in pigs. Leptin mRNA transcripts and protein expression showed the highest expression during early pregnancy and more so on the maternal than the fetal side, again suggesting a more important role around the time of attachment and placentation. These results, along with previous reports from our laboratory on spontaneous fetal loss (implicating the maternal side in fetal arrest) [6, 10, 24] support the current findings that leptin expression may be primarily a maternal response, as opposed to fetal.

Our results show that endometrial *LEP* transcript levels are significantly lower in mid-pregnancy compared to early pregnancy. This contradicts a previous study [31] in humans whereby placental LEP gene expression and proteins were found to increase throughout pregnancy. Potentially in swine, LEP is predominately necessary from the maternal side during the early stages of pregnancy as the developing embryo cannot yet support sufficient production through adipose tissue. Our study only went as far as gd50, where lower LEP and OB-Rb proteins were detected on the fetal side at that point. Had our study investigated later pregnancy, we might have observed a rise in fetal LEP/OB-R towards term as reported by previous studies [25].

Past research has suggested that the expression of OB-Rb is regulated by the amount of LEP and an increase in *LEP* gene expression might lead to both down and up regulation of OB-Rb [21]. In the case of our study, LEP (mRNA and protein) was lower than the OB-Rb (mRNA and protein) no matter the gestational day, tissue type, or the health status of the conceptus. Similar findings were reported by Smolinska et al. [20, 21], whereby greater *OB-Rb* gene expression coincided with lower leptin mRNA expression in the pig CAM. During pregnancy, there is a constant demand for control on metabolic, immunologic, anti-inflammatory, and angiogenic pathways. Too much or too little may offset a pregnancy, creating an environment unstable for proper fetal development. Since LEP is involved in many of these mechanisms, its receptor would be necessary at the maternal-fetal interface to carry out biological actions. Constant levels of OB-Rb are necessary whether LEP is available or not. When or if LEP does become available, the receptors are present to take action. Generally, the more transcripts present result in more functional proteins being translated, creating an increased biological function. However, in the present study, this rule does not appear to apply in the classical sense, likely indicating the presence of post-transcriptional modifications or regulators that alter protein translation.

## Conclusions

In conclusion, the present study demonstrates different patterns of uterine LEP and OB-Rb gene and protein expression levels during the waves of spontaneous conceptus loss in swine pregnancy. Compared to the endometrium of non-pregnant animals, greater *LEP* and *OB-Rb* gene expression in pregnant sows suggest a role for LEP/OB-R in the maintenance of a healthy pregnancy in swine. Additionally, the decline in leptin and receptor proteins at the fetal side of the shared fetal-maternal interface during mid-gestation indicates a more important role for these proteins during early pregnancy, around the time of attachment. Overall, LEP and OB-Rb appear to participate in the health and attachment success of developing porcine embryos. With a continuation of research, a better and more complete understanding of the physiological mechanisms behind spontaneous conceptus loss may help increase litter sizes, resulting in a better economic production of commercial swine.

## Abbreviations

ACTB: β-actin
BSA: Bovine serum albumin
CAM: Chorioallantoic membrane
Endo: Endometrium
gd: Gestational days
H&E: Hematoxylin and eosin
HRP: Horseradish perioxidase
IF: Immunofluorescence
LEP: Leptin
OB-R: Leptin receptor
PCR: Polymerase chain reaction
TBS-T: Tris-buffered saline containing Tween
VEGF: Vascular endothelial growth factor.

## References

[1] Pope WF, Geisert RD, Zavy MT (1994). Embryonic mortality in swine. Embryonic Mortality in Domestic Species.

[2] Stroband H, Van der Lende T (1990). Embryonic and uterine development during early pregnancy in pigs. J Reprod Fert.

[3] Ford SP, Vonnahme KA, Wilson ME (2002). Uterine capacity in the pig reflects a combination of uterine environment and conceptus genotype effects. J Anim Sci.

[4] Geisert R, Schmitt R (2002). Early embryonic survival in the pig: can it be improved?. J Anim Sci.

[5] Vonnahme K, Wilson M, Foxcroft G, Ford S (2002). Impacts on conceptus survival in a commercial swine herd. J Anim Sci.

[6] Tayade C, Fang Y, Hilchie D, Croy BA (2007). Lymphocyte contributions to altered endometrial angiogenesis during early and midgestation fetal loss. J Leukocyte Biol.

[7] Ross JW, Ashworth MD, Stein DR, Couture OP, Tuggle CK, Geisert RD (2009). Identification of differential gene expression during porcine conceptus rapid trophoblastic elongation and attachment to uterine luminal epithelium. Physiol Genomics.

[8] Vallet J, Freking B, Miles J (2011). Effect of empty uterine space on birth intervals and fetal and placental development in pigs. Anim Reprod Sci.

[9] Pope W, First N (1985). Factors affecting the survival of pig embryos. Theriogenology.

[10] Tayade C, Black GP, Fang Y, Croy BA (2006). Differential gene expression in endometrium, endometrial lymphocytes, and trophoblasts during successful and abortive embryo implantation. J Immunol.

[11] Wessels JM, Linton NF, van den Heuvel MJ, Cnossen SA, Edwards AK, Croy BA, Tayade C (2011). Expression of chemokine decoy receptors and their ligands at the porcine maternal–fetal interface. Immunol Cell Biol.

[12] Edwards AK, van den Heuvel MJ, Wessels JM, LaMarre J, Croy BA, Tayade C (2011). Expression of angiogenic basic fibroblast growth factor, platelet derived growth factor, thrombospondin-1 and their receptors at the porcine maternal-fetal interface. Reprod Biol Endocrinol.

[13] Cao Y (2007). Angiogenesis modulates adipogenesis and obesity. J Clin Invest.

[14] Ashworth CJ, Hoggard N, Thomas L, Mercer JG, Wallace JM, Lea RG (2000). Placental leptin. Rev Reprod.

[15] Barb CR, Hausman GJ, Houseknecht KL (2001). Biology of leptin in the pig. Domest Anim Endocrinol.

[16] Tartaglia LA (1997). The leptin receptor. J Biol Chem.

[17] Sierra-Honigmann MR, Nath AK, Murakami C, Garcia-Cardena G, Papapetropoulos A, Sessa WC, Madge LA, Schechner JS, Schwabb MB, Polverini PJ, Flores-Riveros JR (1998). Biological action of leptin as an angiogenic factor. Science.

[18] Bouloumie A, Drexler HC, Lafontan M, Busse R (1998). Leptin, the product of Ob gene, promotes angiogenesis. Circ Res.

[19] Suganami E, Takagi H, Ohashi H, Suzuma K, Suzuma I, Oh H, Watanabe D, Ojima T, Suganami T, Fujio Y, Nakao K, Ogawa Y, Yoshimura N (2004). Leptin stimulates ischemia-induced retinal neovascularization: possible role of vascular endothelial growth factor expressed in retinal endothelial cells. Diabetes.

[20] Smolinska N, Siawrys G, Kaminski T, Przala J (2007). Leptin gene and protein expression in the trophoblast and uterine tissues during early pregnancy and the oestrous cycle of pigs. J Physiol Pharmacol.

[21] Smolinska N, Kaminski T, Siawrys G, Przala J (2009). Long form of leptin receptor gene and protein expression in the porcine trophoblast and uterine tissues during early pregnancy and the oestrous cycle. Anim Reprod Sci.

[22] Smolinska N, Kaminski T, Siawrys G, Przala J (2013). Expression of leptin and its receptor genes in the ovarian follicles of cycling and early pregnant pigs. Animal.

[23] Yoon SJ, Cha KY, Lee KA (2005). Leptin receptors are down-regulated in uterine implantation sites compared to interimplantation sites. Mol Cell Endocrinol.

[24] Wessels JM, Edwards AK, Khalaj K, Kridli RT, Bidarimath M, Tayade C (2013). The microRNAome of pregnancy: Deciphering miRNA networks at the maternal-fetal interface. PLoS One.

[25] Forhead AJ, Fowden AL (2009). The hungry fetus? Role of leptin as a nutritional signal before birth. J Physiol.

[26] Siawrys G, Kaminski T, Smolinska N, Przala J (2007). Expression of leptin and long form of leptin receptor genes and proteins in pituitary of cyclic and pregnant pigs. J Physiol Pharmacol.

[27] Gu T, Zhu MJ, Schroyen M, Qu L, Nettleton D, Kuhar D, Lunney JK, Ross JW, Zhao SH, Tuggle CK (2014). Endometrial gene expression profiling in pregnant Meishan and Yorkshire pigs on day 12 of gestation. BMC Genomics.

[28] Ramos MP, Rueda BR, Leavis PC, Gonzalez RR (2005). Leptin serves as an upstream activator of an obligatory signaling cascade in the embryo implantation process. Endocrinology.

[29] Simon C, Dominquez F, Remohi J, Pellicer A (2001). Embryo effects in human implantation: embryonic regulation of endometrial molecules in human implantation. Ann NY Acad Sci.

[30] Malik NM, Carter ND, Murray JF, Scaramuzzi RJ, Wilson CA, Stock MJ (2001). Leptin requirement for conception, implantation, and gestation in the mouse. Endocrinology.

[31] Lea RG, Howe D, Hannah LT, Bonneau O, Hunter L, Hoggard N (2000). Placental leptin in normal, diabetic and fetal growth-retarded pregnancies. Mol Hum Reprod.

